# Ticks (Acari: Ixodidae) Parasitizing Red Foxes (*Vulpes vulpes*) in Slovakia and New Data About Subgenus *Pholeoixodes* Occurrence

**DOI:** 10.2478/s11686-020-00184-4

**Published:** 2020-03-25

**Authors:** Grzegorz Karbowiak, Michal Stanko, Martina Miterpaková, Zuzana Hurníková, Bronislava Víchová

**Affiliations:** 1 Witold Stefański Institute of Parasitology of Polish Academy of Sciences, Twarda 51/55, 00-818 Warsaw, Poland; 2 Institute of Parasitology, Slovak Academy of Sciences, Hlinkova, 3, 04001 Košice, Slovakia

**Keywords:** *Ixodes crenulatus*, *Ixodes hexagonus*, *Vulpes vulpes*

## Abstract

**Background:**

Distribution and biology of *Pholeoixodes* ticks is not very well understood. The goal of the study was to collect new data on the *Pholeoixodes* tick occurrence in Slovakia.

**Methods:**

Tick infestation of red foxes in the regions of Košice, Prešov, Bratislava and Žilina was studied during the period 2017–2018. Ticks were collected from the fur of animals using tweezers and identified using appropriate keys. In total, 146 red foxes (*Vulpes vulpes*) were investigated.

**Results:**

In total, 39 (26.7%) of animals were found to be infected with ticks from five species. *Pholeoixodes* ticks were found on 13 (3.4%) of the foxes: *Ixodes hexagonus* (Leach, 1815) on 5 specimens (3.4%), in the Košice, Prešov and Žilina regions; *I. crenulatus* (Koch, 1844) on 8 specimens (5.5%) in the Prešov and Bratislava regions; *Ixodes ricinus* (Linnaeus, 1758) collected from 25 (17.2%) foxes in every locality; *Dermacentor reticulatus* (Fabricius, 1794) from 5 foxes (3.4%) in the Košice, Prešov and Žilina regions; *Haemaphysalis concinna* (Koch, 1844), from 4 foxes (2.8%) from the Košice region.

**Conclusions:**

*Ixodes hexagonus* has been previously recorded in Slovakia. However, this is the first finding of *I. crenulatus* in the country. The morphological features of the *I. crenulatus* specimens found in Slovakia were identical to those of ticks described in Poland and descriptions given in identification keys.

## Introduction

The subgenus *Pholeoixodes* (Schulze, 1942), includes tick species occurring in the Palaearctic, Nearctic and Neotropical Zones. They are distinguished from other ticks of the *Ixodes* genus by many morphological features, such as a short *capitulum* and thickset legs. They are also characterised by the presence of relatively short internal spurs on the coxae of the first legs, or in some cases, their complete absence. In addition, the tarsi of the first leg are stepped below Haller’s organ and the genital aperture is located between the coxae 3. While three pairs of setae can be seen on the anal plate of nymphs, five are present in adult females [14, 30, 37]. All *Pholeoixodes* ticks engage in a nest-dwelling mode of feeding, residing in the nests and burrows of their hosts. Hence, *Pholeoixodes* specimens are only occasionally caught by flags and drags, and most of the data on their occurrence has been based on the specimens collected from hosts [30, 37].

Of the *Pholeoixodes* ticks, five species affecting carnivores in Europe have been described: *Ixodes canisuga* Johnston, 1849; *Ixodes* (*Pholeoixodes*) *crenulatus* Koch 1844; *Ixodes* (*Pholeoixodes*) *hexagonus* Leach, 1815; *Ixodes* (*Pholeoixodes*) *rugicollis* Schulze et Schlottke, 1929; *Ixodes* (*Pholeoixodes*) *kaiseri* Arthur, 1957 [13, 30, 37].

### *Ixodes canisuga* (Johnston 1849)

*Ixodes canisuga* is associated with mammals which inhabit burrows. The most commonly infested species are medium-sized Mustelidae and Canidae, such as the red fox (*Vulpes vulpes*) and badger (*Meles meles*), among others [10, 35, 43]. This tick species is also a common parasite of domestic dogs and has been found on cats [25]. The known distribution of this tick ranges from Great Britain, Ireland and France in the West, to Austria and Germany in the East [10, 31, 39, 43], and Portugal to the south [35]. In addition, *Ixodes canisuga* has been observed in the countries of southern Europe, i.e., Hungary, Romania, Croatia, Serbia, Bosnia and Herzegovina, although these have only been recorded as single sightings [9, 13, 16, 27].

### *Ixodes (Pholeoixodes) crenulatus* (Koch 1844)

*Ixodes crenulatus* is the second tick species to be associated with carnivores; however, infestations have also been observed in large insectivores and large rodents [30, 37]. Its documented range includes Poland, Ukraine and Romania [13, 17, 18, 26, 30, 42]. Its southernmost border runs along northern Iran, Afghanistan and Kazakhstan [14, 30, 37].

### *Ixodes (Pholeoixodes) hexagonus* (Leach, 1815)

*Ixodes hexagonus* is associated with hedgehogs, *Erinaceus europaeus* and *E. roumanicus*, as main hosts and the broad spectrum of medium-sized burrow-inhabiting mammals. Apart from hedgehogs, it is commonly found on red foxes and mustelids, including the badger (*Meles meles*), European pine marten (*Martes martes*), stoat (*Mustela erminea)*, European polecat (*Mustela putorius*) and occasionally the otter (*Lutra lutra*) [4, 7, 8]; it has also been found on large rodents, including beavers (*Castor fiber*) [30, 37, 39]. The distribution of *I. hexagonus* covers almost the whole of Europe, ranging from the British Islands and Atlantic coast on the west [31], across all the countries in the central Europe to the eastern border of Romania. The tick is common in Austria, the Czech Republic [6, 7, 39], Poland [18, 19, 24, 45], Slovakia [5, 23, 32] and Germany [10, 33, 43]. Filippova [14] did not demonstrate its occurrence to the east of the River Bug. The range extends to southern Scandinavia in the north and the Mediterranean coast in the south, where it is regarded as a rare tick species [12, 14, 37]. Isolated populations have been found in northern Africa and the Middle East [41, 42].

### *Ixodes (Pholeoixodes) kaiseri* (Arthur, 1957)

*Ixodes kaiseri* has a poorly known range. Its described occurrence covers south-eastern Europe from Romania and Moldavia, extends across southern Ukraine to Kazakhstan to the east, and towards Israel and Egypt to the south [13, 14]. Wodecka et al. [44] report the presence of *Pholeoixodes* females, nymphs and larvae from raccoon dogs and badgers, and these were morphologically distinguished from *I. crenulatus* and *I.* *hexagonus*. An analysis of thirty-four ITS2 sequences revealed high similarity to *I. kaiseri*.

### *Ixodes (Pholeoixodes) rugicollis* (Schulze et Schlottke, 1929)

*Ixodes rugicollis* is rarely observed in Europe, with only isolated observations being recorded in localities in France, eastern Germany, Switzerland, Poland and Romania [8, 13, 33, 37, 42]. Ticks (nymphs and adult females) were collected from dogs in the Podkarpackie voivodeship and from badgers in the Dolnośląskie voivodeship in Poland. However, no detail has been provided regarding their exact coordinates and developmental stage [38].

## Materials and Methods

**Fig. 1:**
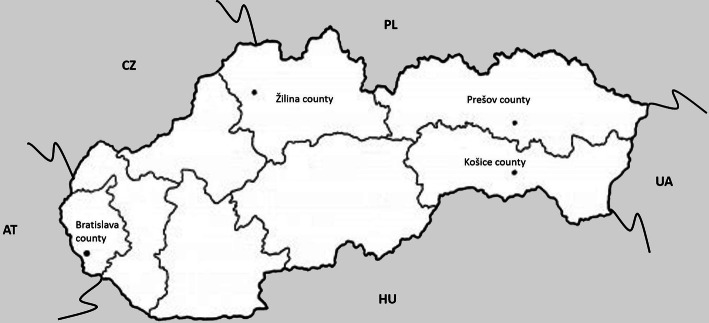
Map of Slovakia with marked counties, where the samples were collected

The present study was conducted in Slovakia during the period 2017–2018, in the regions of Košice, Prešov, Bratislava and Žilina (Fig. 1). Red foxes were legally shot during the monitoring of the effect of oral antirabies vaccination by the State Veterinary and Food Administration of the Slovak Republic. The research was conducted in compliance with the internationally required guidelines under the special permit of the Ministry of Environment of the Slovak Republic. Ectoparasites were collected from animal skin and fur by combing and using tweezers, and preserved in 70% ethanol until further study. The ticks were determined using appropriate keys to distinguish the central and east-European species by Filippova [14], Nosek and Sixl [29] and Siuda [37].

The structure of tick groups were characterised by the prevalence and intensity of infestation, the factors commonly used in the description of parasitocenoses. However, because there is often no correlation between the intensity of infestation and the incidence of parasites, the index of infection (*Z*) is additionally used. *Z* is calculated according to the formula *Z* = (*A* × *B*)/*C*^2^, where: *A* = the number of parasites of the given species, *B* = the number of foxes infested with this same parasite species, *C* = the number of foxes examined in the sample. The index is able to prove the dominant parasite species, following the influent and accessory species in the grouping [3, 11, 20], independent of the number of hosts and parasite population size.

## Results

In total, 146 red foxes (*Vulpes vulpes*) were investigated; 112 from the Prešov region, 9 from the eastern part of the Košice region, 17 from the Bratislava region in western Slovakia, and 8 from the Žilina region in the central northern part of Slovakia. Ticks were found on 39 of the animals tested from the regions: 8 from Žilina, 9 from Košice, 10 from Bratislava, and 12 from Prešov. Five species of ticks were found on the foxes: *Ixodes ricinus*, *Dermacentor reticulatus*, *Haemaphysalis concinna*, *Ixodes hexagonus* and *Ixodes crenulatus* (Table 1).

**Table 1:** Tick infestation of foxes

Fox sampling areas	*Ixodes hexagonus*				*Ixodes crenulatus*				*Ixodes ricinus*				*Heamaphysalis concinna*				*Dermacentor reticulatus*			
	1	2	3	Z	1	2	3	Z	1	2	3	Z	1	2	3	Z	1	2	3	Z
Košice *n* = 9 (9) 100.0%	1	2N	11.1%	0.02	1	1 N	11.1%	0.01	6	3F 2M 1N	66.6%	0.44	1	2N	11.1%	0.02	2	2F	11.1%	0.05
Bratislava *n* = 17 (10) 58.8%					2	5F	11.8%	0.10	7	14F 2M 2N 2L	41.2%	1.40	3	1F 27N	17.6%	0.84	3	3F	17.6%	0.03
Prešov *n* = 112 (12) 10.0%	1	1F 1N	0.9%	0.14	5	13F 8N	4.5%	0.73	6	3F 4M	5.4%	0.29					1	1F	0.9%	0.01
Žilina *n* = 8 (8) 100.0%	3	2N 1L	37.5%	0.14					6	6F 7M	75.0%	1.22								
In total *n* = 146 (39) 26.7%	5	1F 5N 1L	3.4%	0.03	8	18F 9N	5.5%	0.14	25	26F 15M 3N 3L	17.2%	0.76	4	1F 29N	2.8%	0.08	5	6F	3.4%	0.02

The number of foxes investigated is provided (n), the number of foxes infested with ticks (1), the number of ticks (2), the prevalence of infestations (3), the index of infection (Z)

*F* female, *M* male, *N* nymph

Among them, *Pholeoixodes* ticks were found on 13 foxes—*I. crenulatus* on two foxes from Bratislava, one from Košice region and five from Prešov, *I.* *hexagonus* on one specimen from Košice region, one from Prešov and three from Žilina. There were some mixed infestations: *I. ricinus* + *I. crenulatus* on single specimens from Prešov and from Košice; *I. ricinus* + *I. hexagonus* on single specimens from Košice and from Žilina; *D. reticulatus* + *I. hexagonus* on one animal from Košice. There were no mixed infestations with *I. crenulatus* and *I. hexagonus*.

The analysis of prevalence and index of infection *Z* show that the dominant species in all localities was *Ixodes ricinus*—the prevalence of infestation was 5.4–66.6%, mean 17.2%; the index of infection *Z* 0.44–1.40, mean 0.76. The influent species were *I. hexagonus* and *I. crenulatus*, the prevalence was (*I. hexagonus*) 0.9–37.5%, mean 3.4%, *Z* = 0.02–0.14, mean 0.03, and (*I.* *crenulatus*) 4.5–11.1%, mean 5.5%, *Z* = 0.01–0.73, mean 0.14, respectively. Accessory species were *H. concinna* and *D. reticulatus*. *H. concinna* was present in two localities—Košice and Bratislava, the prevalence was 11.1 and 17.6%, mean 2,8%, *Z* = 0.02 and 0.84 respectively. *D. reticulatus* occurred in three localities—Kosice, Prešov, Bratislava, the prevalence was 0.9–17.6%, mean 3.4%, *Z* = 0.01–0.05, mean 0.02.

**Fig. 2:**
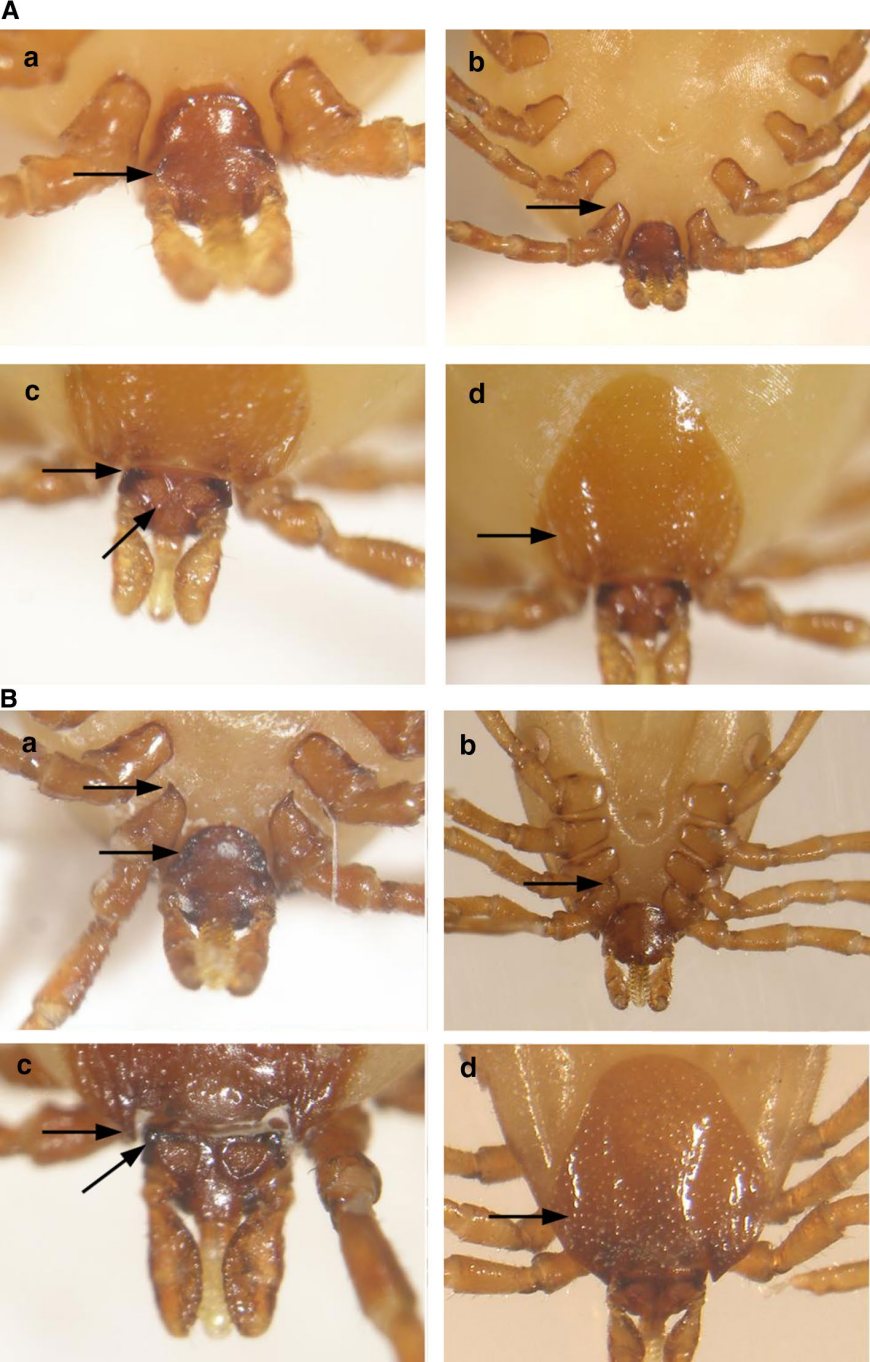
**A** The most important distinguishing morphological features of *Ixodes crenulatus* female: **a** the *auriculae* size; **b** the length of coxae 1 internal spurs with blunt ending; **c** the length and size of *cornua*; **d** deep cervical grooves of scutum and presence of lateral grooves. **B**  The most important distinguishing morphological features of *Ixodes hexagonus* female: **a** the *auriculae* size; **b** the length of coxae 1 internal spurs, with point end and the presence of coxae 2–3 external spurs; **c**— he length and size of *cornua*; **d** deep cervical grooves of scutum

Two species of *Pholeoixodes* ticks were identified. These displayed the morphological features typical of the *Pholeoixodes* subgenus and enabled the species identification, i.e., *I. hexagonus* and *I. cenulatus*. All of these features distinguished the investigated ticks in accordance with all used determination keys, i.e., Filippova [14], Nosek and Sixl [29] and Siuda [37]. The most important features are compiled in Table 2. These are the tarsi of the first leg stepped below Haller’s organ, the genital aperture located between coxae 3 and the relatively short *capitulum*. The *auriculae* size, the length of the *cornua*, and the length of the coxae 1 internal spurs distinguished the males and females of these species, while the *cornua* length, the length of coxae 1 internal spurs, and the presence/absence of coxae 2–3 external spurs distinguished the nymphs (Fig. 2). The morphological nomenclature was used according to Estrada-Peña [12].

**Table 2:** The most important morphological features of *I. hexagonus* and *I. crenulatus*, able the species identification (after: 4, 12–14, 16, 19, 37)

	*Ixodes hexagonus*				*Ixodes crenulatus*			
	Female	Male	Nymph	Larvae	Female	Male	Nymph	Larvae
Hypostome						Narrowed at the apex with a clear indentation at the top	About 2 times shorter than the basis capituli; the widest in the front 1/3 of the length	
Basis capituli	Rectangular; postero-lateral angles slightly salient; ending anterirly as cone-like protuberance			With latero-ventral protuberances	Subrectangular dorsally; postero-lateral angles slightly salient; anteriorlu ending not cone-like			With ventral ridge
Porose areas					Presence of double longitudinal ridges between the porose areas			
Cornua	Present		Present	Absent	Absent		Absent	Absent
Scutum	Nearly as broad as long				widest rather in front of middle			
Cervical grooves	Faint, wavy, reaching posterior border	Faint, strongly divergent			Shallow, wavy, reaching the posterior border	Chiefly visible as elongate divergent depressions		
Lateral grooves	Slight ridge on the antero-lateral borders				Short			
Coxae I	Postero-internal spur distinct and sharp-ended	Postero-internal defined internal spur well defined		Small and short postero-internal spur	Postero- internal spur relatively short and bluntly angled	Postero-internal spurs at most slightly pointed		Postero-internal spur absent
Coxae II						Slight protuberances at the postero-lateral angles	Unarmed	
Coxae III						Slight protuberances at the postero-lateral angles	Unarmed	
Coxae IV						Slight protuberances at the postero-lateral angles	Unarmed	
Median ventral plate		About as broad as long						
Anal grooves	Pointed in front of anus					Rounded in front of anus		

## Discussion

There is the first evidence of *Ixodes crenulatus* in Slovakia. This species had not been recorded before the present study; however, its occurrence has previously been noted in Poland [17, 18], Czech [28] and Ukraine [1, 14]. *Ixodes hexagonus* has been identified in various parts of the country, including the Ondavská Highlands and Tribeč Mountain [5, 23, 28], as well as the south-eastern area [22].

The structure of tick groupings on foxes is typical for the European population of this host. The prevalence of infestation is typical and similar to values noted in the same geographic latitude. The available data in Central Europe show the infestation of foxes with *I. ricinus* from 17.9 to 82.2% [21, 36], with *D. reticulatus* from 24.5 to 27% [18, 40], *I. canisuga* from 4.8 to 35.3% [25, 40], *I. crenulatus* 2.9% [18], *I. hexagonus* 1.8 to 37.5% [18, 34].

Index of dominance *Z* demonstrates, that the dominant species is *I. ricinus* usually, subdominants *Pholeoixodes* ticks. Other tick species have the accessory character. However, the status of influent and accessory species was locally differentiated, i.e., index *Z* shows that in Prešov, *I. crenulatus* (*Z* = 0.73) dominates and *I. ricinus* is an influent species. Near Bratislava the influent species is *H. concinna* (*Z* = 0.10), and this same species is an accessory in the Košice locality (*Z* = 0.02); however, the southern locality of Bratislava is the occurrence area of this species. Other investigated localities lie on the border of *H. concinna* occurrence [29, 37]. On all localities, *D. reticulatus* is the accessory species—however, its occurrence in Slovakiahas changed during last decades; previously, it was classified as a rare species [2].

A review of available literature indicates a difference of opinion regarding whether two tick species are present, i.e., *Ixodes crenulatus* Koch, 1844 and *Ixodes canisuga* Johnston, 1849, or whether they constitute a single species *Ixodes crenulatus*, and concur with the synonym *Ixodes canisuga*. New publications of Estrada-Peña [13] and Guglielmone [15] have postulated the presence of two species, based on a comprehensive review of descriptions, while authors from eastern and central Europe, notably Filippova [14], Siuda [37] and Nowak-Chmura [30], postulate that *I. canisuga* and *I. crenulatus* are synonymous, i.e., a single species. As mentioned above, *I. crenulatus* has mainly been recorded in Poland and Ukraine, while *I. canisuga* has typically been observed in other European countries.

Two other *Pholeoixodes* species that affect carnivores, *I. kaiseri* and *I. rugicollis*, were not found during the study, and have not been recorded in Slovakia previously. However, according to the literature cited above, their described occurrence range covers Slovakia, and their discovery is only a question of time.
